# Brachial plexopathy: a case–control study of the relation to physical exposures at work

**DOI:** 10.1186/s12995-015-0054-9

**Published:** 2015-04-11

**Authors:** Jørgen Riis Jepsen

**Affiliations:** Department of Occupational Medicine, Hospital of South-western Jutland, Østergade 81-83, DK-6700 Esbjerg, Denmark; Center of Maritime Health and Society, Institute of Public Health, University of Southern Denmark, DK-6700 Esbjerg, Denmark

## Abstract

**Background:**

Work-related upper limb disorders constitute a diagnostic challenge. However, patterns of neurological abnormalities that reflect brachial plexus dysfunction are frequent in limbs with pain, weakness and/or numbness/tingling. There is limited evidence about the association between occupational physical exposures and brachial plexopathy.

**Methods:**

80 patients with brachial plexopathy according to defined criteria and 65 controls of similar age and sex without upper limb complaints were recruited by general practitioners. Patients and controls completed a questionnaire on physical and psychosocial work-exposures and provided psychophysical ratings of their perceived exposures. The exposures of cases and controls were compared by a Wilcoxon rank sum test. Odds ratios and dose–response relationships were studied by logistic regression.

**Results:**

Whether assessed as the extent during the workday or days/week, most physical exposures, in particular upper limb posture and repetition, were significant risk indicators with clear dose–response relationships. These findings were supported by psychophysical responses that also identified perceived work pace and the use of force as risk indicators. The identified psychosocial relations were limited to measures reflecting physical exposures.

**Conclusions:**

While the identified risk indicators have previously been associated to upper limb *symptoms* as well as to diagnosed *disorders* other than brachial plexopathy, this study indicates an association between physical and work-exposures and brachial plexopathy. Longitudinal studies should be conducted in order to exclude bias from information and selection, both of which may occur with the applied case–control design.

## Background

In addition to pain, which is often of a neuropathic character, people with upper limb complaints frequently report weakness and/or numbness/tingling. The character of pain may be nagging, stabbing, burning, “like tooth ache” and/or shooting [1]. This combination of symptoms suggests neural involvement and may represent a disorder of the brachial plexus.

Few studies, however, have established a relation between physical exposures and brachial plexopathy [2], although many have suggested hypothetical relations [3-7]. The limited evidence for the existence and frequency of relation to work of brachial plexopathy argues for further research in this field.

Studies of occupational risk indicators for upper limb disorders should rely on both signs and symptoms [8]. Still, epidemiological studies have mainly been restricted to studies of the relation to regional symptoms [9] or to the so-called “specific” disorders such as, e.g. carpal tunnel syndrome [10,11], wrist tendinitis [11,12], epicondylitis [11] and shoulder tendinitis [13,14]. The challenge is that a major group of so-called “non-specific” disorders (that do not meet the criteria for “specific” conditions) are only characterised by symptoms [1]. In a study of upper extremity musculoskeletal complaints, a specific diagnosis was made in only 25% and 16% of patients were not given any diagnosis. The remaining 59% patients (n = 389) were labelled as “non-specific”, i.e. “repetitive strain syndrome”, by the clinician. The pain in this group of patients was equally distributed among neck, shoulder, upper arm, elbow, forearm, and wrist/hand and shared the characteristics of neuropathic pain described above. “Repetitive strain syndrome” was significantly associated with activities with elevated arms, repetitive and extreme movements [1]. In a French population, more than 50% of workers participating in an epidemiologic surveillance system presented non-specific symptoms during the preceding 12 months, and only 13% had a defined upper limb musculoskeletal condition, in particular rotator cuff syndrome, carpal tunnel syndrome and epicondylitis [15].

The application in people with upper limb pain in the primary health sector [16] and in clinical occupational medicine [17-19] of a physical examination consisting of semi-quantitative ratings of selective muscle weakness, sensory deviations from normal in homonymous innervation territories and disturbed mechano-sensitivity of nerve trunks has revealed high frequencies of neurological patterns in accordance with neuropathies with various locations. The clinical applicability and feasibility of this approach, which is based on the classical neurological examination, is indicated by good inter-examiner reliability [17,20-22], construct validity in terms of correlation of physical findings to symptoms [19], and significant interrelations of locations of defined focal neuropathy [23]. The majority of the patients that were subjected to this examination displayed neurological physical patterns in accordance with brachial plexopathy with a prevailing infraclavicular location [16,20]. This observation suggests the importance of identifying, managing and preventing this condition.

Brachial plexopathy remains a controversial diagnosis. Some researchers and clinicians argue that the clinical assessment has a limited validity [24] and that the diagnosis mostly cannot be confirmed by electrophysiological studies [25,26]. They therefore reject the frequency of brachial plexopathy [27] Other researchers and clinicians recognize brachial plexopathy as a common condition [28,29] – also in the context of occupational health [30]. Sheth and Belzberg regard brachial plexopathy as the most underrated, overlooked, misdiagnosed, and difficult to manage peripheral nerve compression in the upper extremity [31].

Clinicians tend to emphasize brachial plexopathy as a condition related to an affliction of the brachial plexus on its passage through the scalene triangle and pay less attention to a compromise more distally, e.g. at the passage of the brachial plexus behind the pectoralis minor muscle (pectoralis minor syndrome) [32,33]. In the clinical setting as well as in epidemiological research distorted diagnostics due to this focus would be unfortunate, in particular in case of the latter location being more common.

Brachial plexopathy can be defined as a combination of symptoms (pain or subjective weakness or sensory abnormalities) and physical findings (weakness in defined muscles, mechanical allodynia at the brachial plexus, and sensory deviations from normal with specific locations). This physical approach permits the examiner to diagnose brachial plexopathy and to define its location precisely [17,20] and accurately [19,23] in a significant number of symptomatic patients, including patients that are diagnostically unclassifiable according to standard diagnostic criteria [17,20,34]. With the application of this diagnostic approach, brachial plexopathy with an infraclavicular location was found to be the most frequent diagnosis in a sample of people with upper limb pain in the primary health sector [16] as well as among patients referred to a department of occupational medicine [19,20].

Such physical diagnostic approach based on symptoms and neurological findings permits the identification of proximally located upper limb neuropathic conditions and makes better sense than alternative diagnostic classification systems in common use [16], e.g. those suggested by Sluiter et al. [35]. It would therefore be essential to include physical neurologic signs that can identify brachial plexopathies in diagnostic classification systems for upper limb disorders.

This study aims to assess the association of brachial plexopathy to physical and psychophysical exposures at work.

## Material and methods

### Study design

This case–control study was based on a case group of people with non-traumatic upper limb pain fulfilling defined criteria for brachial plexopathy and a control group without upper limb complaints. The cases and the controls answered the same questionnaire on work exposures. The distribution of exposures was compared for cases and controls.

### Study subjects

Cases: Out of 277 patients with upper limb pain, of 16–65 years of age and in active occupation that were identified by 21 general practitioners, 169 agreed to participate in the study. Eight patients were excluded due to a history of previous acute trauma, or information on medical conditions that may predispose to upper limb disorders (pregnancy, alcoholism, rheumatoid arthritis, cardiac disease, hypothyroidism, diabetes, amyloidosis, polyneuropathy, vitamin B12 deficiency). The remaining 161 patients were physically examined by a single examiner and classified diagnostically according to previously defined criteria [16].

The neurological part of the physical examination has been previously described in details [17,20]. This examination consisted of manual testing of 17 upper limb muscles, assessment of sensation (light touch, pinprick, vibration 256 Hz) in five homonymously innervated territories of the skin corresponding to the peripheral nerves, and assessment of mechanical allodynia at ten defined nerve-locations [16]. Abnormal sensation was recorded with hypo- as well as hyper-exitability and/or abnormal perception of vibration. Brachial plexopathy was defined in the presence of the following characteristics [16]:

#### Symptoms

Pain, subjective weakness or sensory disturbances in either/or the neck, shoulder, arm, or hand.

#### Brachial plexopathy at the infraclavicular level (pectoralis minor syndrome)

The definition of this condition required all three of the following findings:

- Weakness of the posterior deltoid, biceps brachii, and radial flexor of wrist muscles and one or more of the following muscles: Triceps, short radial extensor of wrist, long extensor of thumb, long flexor of thumb, short abductor of thumb, pectorals, deep flexor to the 5^th^ digit, small adductor of the 5^th^ digit.
- Mechanical allodynia with mild pressure at the infraclavicular brachial plexus on the passage behind the pectoralis minor muscle.
- Sensory abnormalities in the deltoid region.

#### Brachial plexopathy at the supraclavicular level (scalene triangle syndrome)

The definition of this condition required all three of the following findings:

- Weakness of the infraspinatus, posterior deltoid, and biceps brachii muscles. Normal strength in the radial flexor of wrist (unless concomitant more distal neuropathy).
- Mechanical allodynia with mild pressure at the brachial plexus at the passage through the scalene triangle
- Sensory abnormalities in the deltoid region.

**Figure 1:**
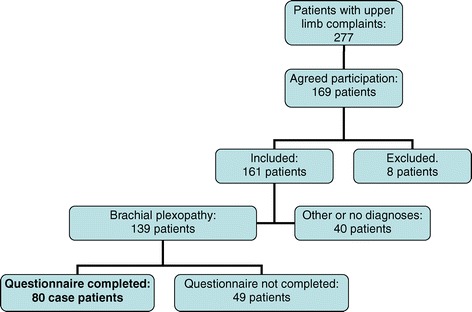
Study sample.

139 patients were diagnosed with brachial plexopathy, and the affliction within the plexus was localized. Based on the same protocol [16] additional upper limb disorders were diagnosed (Figure 1).

Out of patients defined with brachial plexopathy, 80 patients who answered the exposure questionnaire [36] constituted the case group (Figure 1) giving a response rate for cases of 58%. The mean age (SD) of the cases was 43.9 (10.1) years for 61 females and 46.3 (9.1) years for 19 males, respectively.

Controls: 86 control subjects were recruited from the same general practitioners. For each case, the selected control subject was the first subsequent patient after each enrolled case who belonged to the same sex and age-group and who agreed to participate. The controls fulfilled identical inclusion and exclusion criteria as the cases. They could have any occupation and call for any complaint, work-related or not except for the requirement of the absence of any neck or upper limb complaints.

**Figure 2:**
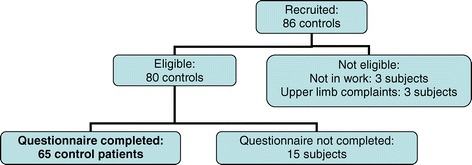
Sample of controls.

Out of these, 65 control subjects who answered the exposure questionnaire [36] constituted the final group of controls (Figure 2) giving a response rate for controls of 81%. The mean age (SD) of the 65 controls was 41.9 (10.4) years for 49 females and 47.3 (9.9) years for 16 males, respectively.

### Assessment of work exposures

A questionnaire was developed for the assessment of physical and psychosocial work exposures and rating of psychophysical perceptions [36]. This questionnaire combined previously published questionnaires, out of which two addressed physical exposures [37,38], one psychophysical items [39] and one psychosocial issues [40]. The final questionnaire included questions to which answers were to be ranked in terms of duration or intensity. Some psychophysical ratings assessed the respondents’ perceptions for each side while others did not (e.g. work pace and back position). All scores were ordinal as reflected by the legends for Tables 1, 2, 3, 4 and 5.

**Table 1:** **Extent of time for various job tasks and exposures according to Wiktorin** [37]

**Exposure**	**U**	**Controls**		**Cases**		**p**
		**N**	**Median**	**N**	**Median**	
A. Sitting (a)	−4.2	65	3	77	1	0.0000
B. Standing (a)	5.3	64	2	80	5	0.0000
C. Walking (a)	3.1	63	2	78	3	0.0023
D. Operating a computer (a)	−3.7	64	2	80	1	0.0003
E. Work with vibrating hand tools (a)	2.1	64	1	79	1	0.0385
F. Work with arms in or over shoulder level (a)	3.4	65	1	80	1	0.0006
G. Work with stretched arms ≥ 45 degrees forward or laterally (a)	3.6	63	1	80	2	0.0004
H. Work with almost maximally flexed neck (a)	3.0	63	1	79	2	0.0025
I. Work with identical arm movements many times per minute (a)	4.4	64	1	79	3	0.0000
J. Work with identical hand/finger movements many times/minute (a)	2.7	64	2	79	3	0.0074
K. Precision work > 2 hours/day (b)	1.2	65	1	80	1	0.2441
L. Work with arms in or over shoulder height > ½ hour/day (b)	3.5	65	1	80	1	0.0004
M. Work with arm extension ≥ 45 degrees forward or laterally > ½ hour/day (b)	3.3	63	1	78	4	0.0010
N. Work with identical arm movements many times/minute > 2 hours/day (b)	4.8	63	1	80	5	0.0000
O. Work with identical hand or finger movements many times/minute > 2 hours/day (b)	2.6	65	1	79	5	0.0088
P. Fingers exposed to cold (b)	2.9	65	1	76	1	0.0041

The questions read: (a) How big a proportion of a normal work-day did you …..? Answers on a five step scale: Almost never/not at all (1), approx. 1/4 of the time (2), approx. 1/2 of the time (3), approx. 3/4 of the time (4), almost all the time (5). (b) Did you have………? Answers on a five step scale: Almost never/not at all (1), 1–3 days/months (2), 1 day/week (3), 2–4 days/week (4), every day (5).

U = test magnitude. p = significance level.

Wilcoxon Mann–Whitney rank sum test.

**Table 2:** **Odds ratios depending of the temporal extent of exposure during the workday according to Torgén et al**. [38]

**Risk factor (Proportion of workday)**	**Controls(N)**	**Cases (N)**	**OR (95% CI)**
**Sitting**			
Almost never/not at all	11	42	1.00
Approx. ¼ - ½ of workday	27	18	0.54 (0.24 1.21)
More than ½ of workday	27	17	0.18 (0.05-0.71)
**Standing**			
Almost never/not at all	20	11	1.00
Approx. ¼ - ½ of workday	31	18	1.11 (0.44-2.82)
More than ½ of workday	13	51	7.49 (2.90-19.37)
**Walking**			
Almost never/not at all	18	16	1.00
Approx. ¼ - ½ of workday	35	32	1.02 (0.46-2.25)
More than ½ of workday	10	30	3.33 (0.69-7.15)
**Operating a computer**			
Almost never/not at all	23	54	1.00
Approx. ¼ - ½ of workday	24	15	0.28 (0.12-0.62)
More than ½ of workday	17	11	0.29 (0.123-0.71)
**Extent of work with vibrating hand tools**			
Almost never/not at all	63	71	1.00
Approx. ¼ of workday or more	1	8	7.11 (0.87-58.42)
**Extent of work with arms in or over shoulders**			
Almost never/not at all	51	43	1.00
Approx. ¼ - ½ of workday	13	25	2.28 (1.04-4.99)
More than ½ workday or more	1	12	14.23 (1.77-113.91)
**Extent of work with arms extended more than 45** ^**0**^ **forward or to the side**			
Almost never/not at all	37	27	1.00
Approx. ¼ - ½ of workday	21	26	1.79 (0.84-3.80)
More than ½ of workday	2	18	7.80 (2.67-22.80)
**Extent of work with almost maximal neck flexion**			
Almost never/not at all	44	39	1.00
Approx. ¼ - ½ of workday	17	22	1.49 (0.69-3.19)
More than ½ of workday	1	10	10.35 (2.26-47.37)
**Extent of work with identical arm movements many times per minute**			
Almost never/not at all	41	24	1.00
Approx. ¼ - ½ of workday	13	22	2.84 (1.22-6.62)
More than ½ of workday	10	33	5.54 (2.34-13.15)
**Extent of work with identical hand/finger movements many times per minute**			
Almost never/not at all	25	19	1.00
Approx. ¼ - ½ of workday	21	22	1.36 (0.59-3.14)
More than ½ of workday	18	38	2.74 (1.22-6.16)

Logistic regression.

**Table 3:** **Odds ratios relating to the amounts of workdays for various job tasks and exposures according to Torgén et al.** [38]

**Risk factor (Amount of days with job tasks and exposures)**	**Controls (N)**	**Cases(N)**	**OR (95% CI)**
**Precision work > 2 hours/day**			
Almost never/not at all	54	61	1.00
1-3 days/month	3	2	0.43 (0.15-1.30)
1 day/week	2	1	0.26 (0.03-2.02)
2-4 days/week	1	3	0.19 (0.01-2.62)
Every day	5	13	1.15 (0.10-13.88)
**Working over shoulder level > ½ hour/day**			
Almost never/not at all	51	42	1.00
1-3 days/month	1	3	3.64 (0.37-36.32)
1 day/week	4	3	0.91 (0.19-4.30)
2-4 days/week	4	6	1.82 (0.48-6.88)
Every day	5	26	6.31 (2.23-17.87)
**Working with extended arms > 45** ^**0**^ **or more forward or laterally > ½ hour/day**			
Almost never/not at all	39	26	1.00
1-3 days/month	2	4	2.92 (0.50-17.09)
1 day/week	3	1	0.49 (0.05-4.94)
2-4 days/week	2	9	6.59 (1.32-32.82)
Every day	17	38	3.27 (1.55-6.91)
**Working with identical arm movements many times/minute > 2 hours/day**			
Almost never/not at all	45	27	1.00
1-3 days/month	4	1	0.42 (0.04-3.92)
1 day/week	2	3	2.50 (0.39-15.93)
2-4 days/week	4	6	2.50 (0.65-9.66)
Every day	10	43	7.17 (3.10-16.56)
**Working with identical hand/finger movements many times/minute > 2 hours/day**			
Almost never/not at all	34	25	1.00
1-3 days/month	2	3	1.96 (0.31-12.60)
1 day/week	1	2	2.62 (0.22-30.43)
2-4 days/week	6	6	1.31 (0.38-4.53)
Every day	22	43	2.56 (1.24-5.27)
**Fingers exposed to cold**			
Almost never/not at all	55	50	1.00
1-3 days/month	5	5	1.02 (0.28-3.72)
1 day/week	3	1	0.33 (0.03-3.37)
2-4 days/week	0	4	-
Every day	2	16	8.15 (1.78-37.15)

Logistic regression.

**Table 4:** **Psychophysical measures according to Punnett et al.** [39]

**Exposure**	**U**		**Controls**				**Cases**				**p**	
			**N**		**Median**		**N**		**Median**			
	**Right**	**Left**	**Right**	**Left**	**Right**	**Left**	**Right**	**Left**	**Right**	**Left**	**Right**	**Left**
Work pace (a)	2.925		65		6		80		8		0.0034	
Back positions (b)	4.319		65		3		80		5		0.0000	
Neck positions (b)	4.505		64		3		79		5		0.0000	
Shoulder positions (b)	5.235	4.958	65	65	3	2	80	80	6	5	0.0000	0.0000
Arm positions (b)	4.651	5.304	65	65	3	2	80	80	6	5	0.0000	0.0000
Hand positions (b)	3.625	4.211	65	65	4	2	80	80	5.5	5	0.0000	0.0003
Whole body vibration (c)	1.093		64		0		80		0		0.2743	
Local vibration (c)	1.942	1.848	65	65	0	0	80	79	0	0	0.0521	0.0646
Applied hand force (d)	4.746	4.993	65	65	2	2	80	80	6	5	0.0000	0.0000
Applied hand force in uncomfortable positions	4,211	4.281	65	65	1	1	76	76	2	1	0.0000	0.0000
Pressure on hand or arm (d)	3.976		65		0		78		0		0.0001	
Usual weight with manual handling (e)	2.094		33		4		63		5		0.0362	
Heaviest weight with manual handling (e)	1.228		32		6.5		62		7.5		0.2194	
Weight of usual tool (e)	1.503		24		2		36		3		0.1329	
Balance of usual tool (f)	1.824		24		1		36		2.5		0.0681	
Force required for holding and using tool (g)	2.901		24		1.5		35		5		0.0037	

Wilcoxon Mann–Whitney rank sum test. All ordinal measures on a VAS-scale 0–10.

(a) Very slow - too fast, (b) (Very comfortable - very uncomfortable, (c) None - a lot, (d) None/very small - very large, (e) Very light – very heavy, (f) Perfect – major imbalance, (g) Very small – very large. NB Calculations for perceptions related to the limbs made for each side.

U = test magnitude. p = significance level.

**Table 5:** **Psychosocial exposure measures according to Søndergaard** [40]

**Psychosocial exposure**	**U**	**Controls**		**Cases**		**p**
		**N**	**Median**	**N**	**Median**	
A. Required to work at high pace (a)	2.9	61	3	77	2	0.003
B. Influence on decisions about the work (b)	−2.8	63	2	77	3	0.005
C. Possibilities for learning (b)	−2.8	63	2	79	3	0.005
D. Influence on the content of work (b)	−2.1	63	3	78	3	0.038
E. Support from immediate superior (a)	−2.6	63	2	75	3	0.009
F. Frequency of consultation with superior of quality of job performance (a)	−2.4	63	3	75	4	0.015
G. Conflict solving skills with superior (b)	−2.0	63	3	75	3	0.047
H. Satisfied with physical work environment (c)	−4.5	53	2	77	2	0.000
I. Satisfied with the way your skills are used (c)	−3.4	63	2	76	2	0.001
J. Self-rating of health (d)	−4.6	65	2	79	3	0.000
K. Happy and satisfied during the last four weeks	−2.3	64	2	79	3	0.024
L. Enthusiastic and full of life during the last four weeks	−3.6	64	3	78	4	0.000
M. Full of energy during the last four weeks	−3.8	64	3	77	4	0.000
N. Warn during the last four weeks	3.5	64	5	78	4	0.000
O. Tired during the last four weeks	2.4	64	5	79	4	0.01

Wilcoxon Mann–Whitney rank sum test.

(a) Always, frequently, at times, rarely, never/almost never, (b) to very high degree, to high degree, partly, to small degree, to very small degree, (c) very satisfied, satisfied, unsatisfied, very unsatisfied, (d) excellent, very good, good, not so good, bad, (e) All the time, most of the time, a lot of the time, some times, little of the time, never.

U = test magnitude. p = significance level.

Various ways to validate these questionnaires have been previously applied. The ability to predict symptoms and the usefulness of psychophysical perceptions for preventive purposes was demonstrated for the questionnaire based on psychophysical items. One of the questionnaires on mechanical exposure was shown to be reliable in use [38]. The advantages of combining questions relating to the exposure and psychophysical perceptions has been documented [41].

Questions regarding psychosocial issues were included in order to assess their potential role as confounders rather than individual risk indicators for brachial plexopathy.

### Statistics

The presence of brachial plexopathy on any or both sides was compared to the reported exposures. The relation between case status and individual ordinal scores for exposures according to Wiktorin et al. [37] was analysed by a Wilcoxon Mann–Whitney rank sum test (Table 1).

In addition, the magnitude of risk expressed by the odds ratios for selected mechanical exposures according to Torgén et al. [38] was calculated with logistic regression and the dose–response relationships estimated. For this calculation, the influence of the proportion of the workday with exposure to each risk factor was assessed with three exposure levels, except for work with vibrating hand tools, which was assessed dichotomously (Table 2). The effect of the number of exposure days per week was assessed with five exposure categories (Table 3). All exposure categories were compared to “Almost never/not at all”.

A Wilcoxon Mann–Whitney rank sum test was also applied for the comparison of the psychophysical ratings [39] in between cases and controls. When applicable, separate analyses were made for the right and left side.

Data were processed by Stata ver. 12.1.

### Ethics

The study complied with the Helsinki declaration. The clinical examination protocol was approved by the local ethics committee (De Videnskabsetiske Komittéer for Region Syddanmark) and signed informed consent was obtained from all participants.

## Results

According to the defined criteria, 9 and12 limbs were diagnosed as right and left supraclavicular brachial plexopathy, respectively, with bilateral affliction in five. 52 and 37 limbs were diagnosed as right and left infraclavicular brachial plexopathy, respectively, with bilateral involvement in 12. Nine and eight patients had a combination of supraclavicular and infraclavicular plexopathy on the right and left side, respectively.

According to the applied diagnostic criteria [16], a high proportion of cases had additional non-neuropathic diagnoses with the major concomitant diagnoses on the right/left side being rotator cuff disorder (12/8), and epicondylitis (19/12), respectively [16]. Simultaneous presence of epicondylitis and rotator cuff disorder was identified in one right limb. All except one limb with brachial plexopathy had additional peripheral neuropathy, which was mainly involving the median and the radial/interosseous nerves at elbow level.

### Relation between cases and controls in terms of ordinally grouped individual exposures, exposures grouped into indices and psychophysical ratings

Assessment of the daily amount of exposure by the application of the mechanical exposure questions by Wiktorin [37] showed that sitting and computer work appeared to be protective in terms of brachial plexopathy. Working with arms at or over shoulder height, stretched arms 45° or more forward or laterally, neck flexion and repetitive arm and hand/finger work all constituted highly significant physical risk exposures while vibrating hand tools had a relatively minor influence on the prevalence of brachial plexopathy (Table 1).

The extension of work tasks with the limb extended more than 45° forward or laterally more than a half hour/day and of repetitive arm, hand or finger work more than 2 hours/day was also strongly associated to case status. Cold exposure was also a risk factor. Precision work exceeding 2 hours/day was not significantly related to brachial plexopathy (Table 1).

**Figure 3:**
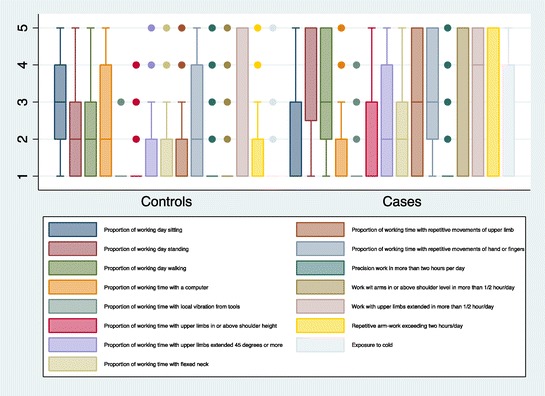
Boxplot illustrating the distribution of the extent of job tasks and exposures in Table 1. The boxes illustrate the interquartile range. The horizontal line represents the median. The vertical lines illustrate the maximal and minimal values excluding outliers, and the dots represent outliers.

The distribution in controls and cases of the extent of the various job tasks is illustrated in the box plot in Figure 3.

The risk estimates for the various job tasks and exposures according to Torgén et al. [38] as illustrated in Table 2 (Proportion of workday exposed) and Table 3 (Number of days/week exposed) showed similar results. There was a dose–response relationship with increasing exposure during the workday for standing, arms in or over shoulder height, arms extended more than 45° forward or laterally, almost maximal neck flexion, identical arm movements many times/minute, and identical finger movements many times/minute. At the maximal exposure levels, the risk estimates for all these variables were significant. There was an increasing risk with increasing amount of walking and work with vibrating hand tools, but the risk estimates did not reach significance. Again, sitting and computer work appeared to be protective in this sample (Table 2).

Except for precision work exceeding 2 hours/day, the analyses of the influence of the amount of days/week exposed showed a similar picture with significantly elevated risk estimates for work over shoulder level more than a half hour/day, arms extended more than 45^0^ forward or laterally more than a half hour/day, identical arm movements many times/minute exceeding 2 hours/day, and identical finger movements many times/minute during more than 2 hours/day. Exposure to cold was also a significant risk factor. There was a dose–response relationship to the number of days exposed per week for all exposures except precision work (Table 3).

All psychophysical measures [39] that relate to work pace, postures, the use of force, the usual weight of manually handled items, and local pressure were significantly related to case status. Local vibration was borderline not significantly related to brachial plexopathy. Whole body vibrations, the weight and balance of the usual tool and the weight of the heaviest handled items were unrelated to case status (Table 4).

Psychosocial exposures [40], for which the distribution differed significantly between cases and controls are illustrated in Table 5. The distribution was the same in the two samples for all other variables (emotional demands, meaning and commitment, collaboration and leadership, interaction between the individual and work, stress and depression).

## Discussion

The high frequency of brachial plexopathies in the original sample of patients from which the cases for this study was drawn [16] is in accordance with previous studies [16,19,20]. With the applied diagnostic definition [16], this study has clearly indicated a number of risk indicators for brachial plexopathy. Whether based on assessments of physical exposures or of psychophysical perceptions, highly significant relations were demonstrated for adverse upper limb postures, repetitive work, work pace and the use of strength.

Two of the identified risk factors, walking and exposure of the fingers to cold (Table 1), can hardly be explained in terms of pathophysiological mechanisms, but may rather be related to the character of work, which may combine walking and cold exposure with other identified risk indicators.

Surprisingly, two exposures were more prevalent among controls than cases: sitting and computer work. The effect of sitting may be explained by the fact that most sitting work does not involve adverse positions and repetitive work, although undertaking sitting tasks that do involve these exposures may in fact contribute to further adverse upper limb postures, e.g. by requiring further arm elevation than working in the standing position. Brachial plexopathy has been demonstrated in a series of patients with intensive computer work [42], and neurological patterns in accordance with brachial plexopathy were common in symptomatic computer operators in active occupation [43]. A meta-analysis found moderate evidence for the relation between computer use and upper limb pain but limited relation to specific musculoskeletal disorders out of which brachial plexopathy was not addressed [44]. The current findings might be explained by an exposure to computer work of a low intensity, which does not constitute a risk for brachial plexopathy.

The relation between local vibration and upper limb nerve afflictions is well documented [45], but the effect on brachial plexopathy in the current study was limited. The minor impact of vibration may relate to the distance between the hand and the brachial plexus. A more likely explanation, however, is the limited number of subjects in the sample that were exposed to local vibration (Table 2).

The analyses assessed the association between the exposures and brachial plexopathy with a supraclavicular as well as an infraclavicular location. Due to the dominance of infraclavicular locations in this sample, the conclusions concern infraclavicular rather than supraclavicular brachial plexopathy. This observation demonstrates the importance of studying the brachial plexus at several locations – not only at the scalene triangle.

The assessment of exposure by a questionnaire based on questions developed and validated by others included physical, psychophysical, and psychosocial issues. Although a questionnaire approach to exposure assessment is generally regarded as inferior to direct observations, the former has major advantages in terms of costs and practical execution. A French study found that the questionnaire was superior to direct observations in identifying workers at high risk of upper limb work-related musculoskeletal disorders [46]. The inclusion of the psychophysical questions was regarded advantageous due to the ability of these to qualify questions addressing physical exposures [41]. The ability of the physical and psychophysical ratings to identify comparable risk indicators supports the consistency of the presented findings.

### Validity

Non-experimental research has a number of innate methodological constraints. One point of importance is the potential confounding that may occur when the frequency of risk indicators other than those studied differs in between cases and controls. This potential for bias is an acknowledged weakness of the case–control design.

#### Bias of information

The collection on a cross-sectional basis of questionnaire information on exposure may result in information bias. Such bias would be differential and lead to spuriously increased risk estimates if, compared to controls, cases would overrate their exposures. The absence of differential misclassification has been demonstrated for some of the posed questions [37] while the risk for differential misclassification cannot be excluded in the remaining questions on mechanical exposure [37,47]. It has, however, been shown that questions addressing physical risk exposures for upper limb disorders seem to cause less misclassification than is the case for low back disorders [48]. In this context, the absence of a relation between symptoms and exposures, which the responders would be likely to regard as related to upper limb symptoms (e.g. precision work or computer work) is noteworthy. Information bias cannot therefore be excluded but is regarded as less likely to seriously distort the results.

#### Bias of selection

The selection of the studied samples of patients and controls is another potential source of bias. In spite of instructions, the general practitioners may not have selected all eligible patients. For example, although the practitioners were asked to enrol as cases all patients with non-traumatic upper limb symptoms, there could be a reluctance to enrol those with milder or easily interpreted symptoms rather than patients for which they may have been more inclined to request a second opinion from the research team [16]. Bias from the selection of controls can also not be excluded. While the general practitioner was asked to select as control subject the first eligible patient who was willing to complete the questionnaire, one cannot be sure if this really happened. Whether the occupations differed in between cases and controls is not known. A higher share of manual workers in the case group could induce a bias of selection. Consequently, although the response rate and the distribution on age and sex were comparable in cases and controls, a potential differential selection may have influenced the composition of the final sample of controls.

### Psychosocial issues

It is noticeable that answers to questions about psychosocial issues [40] that differed between cases and controls (Table 5) reflect either the physical exposure or conditions that can either be linked to circumstances that characterize the physical work-environment such as repetitive work (Tables 1, 2 and 3), or the psychophysical perceptions, e.g. the requirement of working at high pace (Table 4-A). Most responses to psychosocial issues, however, were not linked to these factors. This applies for, e.g. the perceived emotional demands from work, the meaning and commitment of work, the collaboration and leadership, the interaction between the individual and the work, as well as for perceptions that reflect stress and depression.

This study supports previous longitudinal studies that have shown evidence for a link between upper limb symptoms and the influence of high work demands [49], little control at work [49], and low job satisfaction [50]. The adverse influence of little job control and additionally of supervisor support has also been demonstrated in a cross-sectional study that, however, estimated the magnitude of the population attributable fraction of upper limb disorders for exposure to psychological factors to 12% [51]. While several physical risk indicators for arm pain were identified, no psychosocial stressors were identified in a case–control study of patients presenting to primary care, in which, however, a number of mental health variables were linked to case status [52].

High work demands, little control, low job satisfaction, and supervisor support are common terms in trades with repetitive and paced work with adverse upper limb positions. Consequently, the demonstration in the present study of the role of these psychosocial exposures is not surprising. On the other hand, the absence of an influence of psychosocial exposures that are not linked to physical risk indicators suggests psychosocial factors in themselves to be less likely risk indicators for brachial plexopathy. This observation supports the validity of the identified relations to mechanical exposures.

### Physical examination and case-definition

With a high inter-observer reproducibility, a detailed neurological examination of patients with and without upper limb symptoms has indicated frequent neurological patterns in accordance with brachial plexopathies [19,20]. In a sample of people with upper limb pain and people with other disorders, this examination predicted with a high accuracy the presence or absence of symptoms [19]. The neurological items studied in this validity study were comparable to those included in the current definitions of brachial plexopathy. This definition permitted the diagnosis of brachial plexopathy in a high number of people with upper limb pain in clinical occupational medicine [19] and general practice [16].

Still, the endpoints in terms of defined disease outcome may be challenged. Brachial plexopathy is a controversial diagnostic construct, and there is neither consensus about its diagnosis, frequency nor potential work-relatedness. The main challenge is the absence in most brachial plexopathies of electrophysiological abnormalities [25], which tends to be interpreted as its nonexistence. This is unfortunate because brachial plexopathy should be diagnosed based on clinical findings rather than electrophysiological studies [53]. Although the latter are regarded by many as the “gold standard” for peripheral neuropathy, a mixed and partial nerve affliction with few myelinated fibers intact and reinnervation taking place may result in entirely normal findings [54].

Characteristic symptoms in these patients include pain, which is often of a neuropathic character, weakness and/or numbness/tingling. The content of the applied neurological examination was derived from anatomical facts and pathophysiology that has led to an established paradigm on which the traditional neurologic bedside examination is based: Focal neuropathy with a certain location is likely to result in rather specific neurological deviations from normal. These abnormalities may well be minor and include weakness and sensory deviations from normal distal to the nerve affliction, and a maximal mechanical allodynia of nerve trunks where, according to the neurological patterns, the affliction is located.

The case definition for infraclavicular brachial plexopathy (pectoralis minor syndrome) that was applied in this study is based on anatomical facts. A compromise at this location is likely to involve the lateral-most portion of the brachial plexus because the available space for passage behind the pectoralis minor muscle is more limited laterally than medially. This portion of the brachial plexus contains neurons from the axillary, musculocutaneous and radial nerves that supply motor innervation to the deltoid, biceps, and radial flexor of wrist muscles as well as the cutaneous axillary nerve innervation of the deltoid region. With a supraclavicular location of brachial plexopathy (scalene triangle syndrome), the neurons supplying the suprascapular nerve that innervates the infraspinatus muscle are at risk because of the location in the top of the scalene triangle.

The applied diagnostic criteria for brachial plexopathy cannot be satisfied with any other upper limb condition. On the other hand, when these criteria are met, concomitant disorders of a neuropathic or a non-neuropathic character may also be present. Concomitant disorders, in particular rotator cuff disorders, epicondylitis, and peripheral upper limb neuropathies were frequent in the studied sample [16]. Their relative influence as dependant variables cannot be determined in this study. It can, however, be argued that rotator cuff disorders and epicondylitis may share similar risk indicators and that these conditions may complicate or predispose to brachial plexopathy. This study cannot determine the potential relation and succession of such events.

It is noticeable that the criteria for diagnosing a concomitant peripheral neuropathic condition were fulfilled for all except one limb with brachial plexopathy. This observation is in accordance with clinical experiences as well as with the “double crush” theory [55], which can explain neurological patterns and symptoms that may appear confusing to the clinician. According to the diagnostic criteria applied in this study [16], the identification of a peripheral neuropathic condition required a defined combination of motor and sensory outcomes as well as allodynia with gentle palpation of the nerve trunk at the location of entrapment. The latter suggests a peripheral nerve involvement at this specific location.

The correlation of regional pain to physical findings in the same region characterises much analytical research targeting upper limb disorders. However, epidemiological studies that aim to correlate the location of symptoms with physical findings at the same location may be distorted because upper limb pathology may be situated at a distance to the dominant location(s) of symptoms such as pain. This is particularly true with involvement of the peripheral nerves (referred pain [56]). Upper limb symptoms are often of a fluctuant character and intensity, and their locations are likely to change and spread. Consequently, the physical examination should include the whole limb as well as the neck and include a careful examination of the peripheral nervous system [57]. Such comprehensive physical approach can identify proximally located neuropathic conditions such as at the brachial plexus, and explain the fluctuating symptoms by a dynamic compression. In the absence of such examination, the correlation between exposures and symptoms with certain locations is likely to be diluted.

### Causality

Viewing the findings of this study in the context of the considerations of causality by Bradford Hill [58], it can be positively concluded that the documented associations are strong, dose–response related, and that the time-relation is clear through the design. The relation between brachial plexopathy and the identified physical exposures and psychophysical perceptions is biologically plausible and there is evidence for the relation of physical findings in the examination of the peripheral nerves to minor or major impacts on nerve tissue. In a literature review, Rempel et al. highlighted the cascade of effects resulting from low level nerve pressure, consisting of inhibition of intraneurial microvascular blood flow, axonal transport and nerve function as well as endoneurial oedema and displacement of myelin. Higher pressure have more profound effects including demyelination, inflammation, fibrosis, growth of new axons and remyelination [59]. Schmid et al. have reviewed the pathophysiological mechanisms [60] and shown that even mild nerve compression in animals is sufficient to induce intraneural inflammation, which is associated with neuropathic pain behaviour [61].

Still, some weak points should be mentioned. Consistency in terms of similar results from other studies is lacking as no other studies have yet demonstrated the relation of brachial plexopathy to mechanical exposures at work. The specificity in terms of causation may also be questioned. A variety of exposures were attributed to brachial plexopathy. In addition, other upper limb conditions may share the risk indicators as here reported for brachial plexopathy. Consequently, this study has demonstrated associations to work exposures but cannot conclude about causality, which needs to be examined in prospective studies of patients with brachial plexopathy without other concomitant disease. In addition, the aggregated effects of the various mechanical exposures, and the interaction in between them should be studied. Most importantly, the demonstration of a reduced occurrence of disease following elimination or reduction of the identified risk exposures would represent an important step for improved prevention of brachial plexopathy.

## Conclusions

This study has demonstrated several adverse physical exposures at work, in particular upper limb posture and repetitive movements of the arm, hand and fingers to be risk indicators for brachial plexopathy with significant dose–response relationships. These findings were supported by psychophysical responses that additionally indicated work pace and the use of force as risk indicators. The identified relations to psychosocial measures were limited to those reflecting physical exposures.

This study has two major clinical implications. With acceptance of the applied diagnostic criteria, brachial plexopathy is a frequent condition, which should not be overlooked. A simple screening of the upper limb nerves can exclude this condition [62]. The diagnosis of brachial plexopathy can provide directions for management and, taking into account the demonstrated risk indicators, for preventive interventions at workplaces.

It can therefore be concluded that this study provides evidence of a relation of mechanical work-exposures to brachial plexopathy. The identified risk indicators for upper limb disorders have previously been associated to *symptoms* as well as to diagnosed “specific” upper limb *disorders* other than brachial plexopathy. Longitudinal studies should be conducted in order to provide evidence of causality and for excluding bias from information and selection, both of which may occur with the applied case–control design.
